# Prevalence of surgical site infections after open reduction and internal fixation for mandibular fractures: a systematic review and meta-analysis

**DOI:** 10.1038/s41598-023-37652-6

**Published:** 2023-07-10

**Authors:** Evangelos Kostares, Georgia Kostare, Michael Kostares, Maria Kantzanou

**Affiliations:** 1 Department of Microbiology, Medical School, National and Kapodistrian University of Athens, 115 27 Athens, Greece; 2 National and Kapodistrian University of Athens Faculty of Medicine: Ethniko kai Kapodistriako Panepistemio Athenon Iatrike Schole, 115 27 Athens, Greece

**Keywords:** Oral diseases, Trauma

## Abstract

Our study aims to estimate the prevalence of surgical site infections (SSI) following open reduction and internal fixation (ORIF) for mandibular fractures and to determine the effect of potential moderators on it. A systematic literature search (Medline and Scopus databases) was conducted independently by two reviewers. The pooled prevalence with 95% confidence intervals was estimated. Quality assessment as well as outlier and influential analysis were performed. Additionally, subgroup and meta-regression analysis were conducted in order the effect of categorical and continuous variables on the estimated prevalence to be investigated. In total, seventy-five eligible studies (comprising a sum of 5825 participants) were included in this meta-analysis. The overall prevalence of SSI following ORIF for mandibular fractures was estimated as high as 4.2% (95% CI 3.0–5.6%) with significant heterogeneity between studies. One study was identified to be critically influential. In the subgroup analysis, the prevalence was 4.2% (95% CI 2.2–6.6%) among studies conducted in Europe, 4.3% (95% CI 3.1–5.6%) among studies conducted in Asia and higher among those conducted in America (7.3%) (95% CI 4.7–10.3%). It is important for healthcare professionals to be aware of the etiology of these infections, despite the relatively low rate of SSI in these procedures. However, further, well-designed prospective and retrospective studies need to be conducted in order this issue to be fully clarified.

## Introduction

The fractures of the mandible are one of the most common types of facial traumas requiring surgical intervention^1^. They can be related to several types of injuries such as assaults and accidents^2,3^ and are mostly noticed in middle-aged males^4^. The most common fracture site is the condyle, accounting approximately for 25.0% to 35.0% of all mandibular fractures^4^. Severe fractured cases could be led to life-threatening situations such as airway obstruction and major hemorrhage^5^ and therefore, should be recognized and treated immediately.

The treatment is usually performed by oral and maxillofacial surgeons (OMFS) or other relevant surgical specialties in hospital settings, and can be either, closed, or open reduction and internal fixation (ORIF)^2,3,5,6^. ORIF is generally considered both effective and safe procedure. Yet, various perioperative events, such as inferior alveolar nerve injury, temporomandibular joint disorders, hemorrhage, surgical site infection (SSI), nonunion of the osseous segments, bone necrosis, soft tissue injury, malocclusion, abscess, and hardware exposure may occur^7–10^.

Surgical site infection (SSI), defined by CDC as a nosocomial infection following a surgical procedure that occurs near the surgical site within 30 days following surgery (or up to 90 when a medical implant is involved), is associated, according to several studies^11,12^, with significant poor surgical outcome as well as considerable personal and health care cost^13^. SSI can be classified as superficial incisional, deep incisional, and organ/space^11^. The reported rate of SSI following ORIF for mandibular fractures varies considerably in the scientific literature^14–17^. Therefore, the aim of the current study is to report a more precise estimation of the prevalence of SSI after ORIF for mandibular fractures, by meta analyzing the available data from the scientific literature.

## Methods

### Search strategy

**Figure 1:**
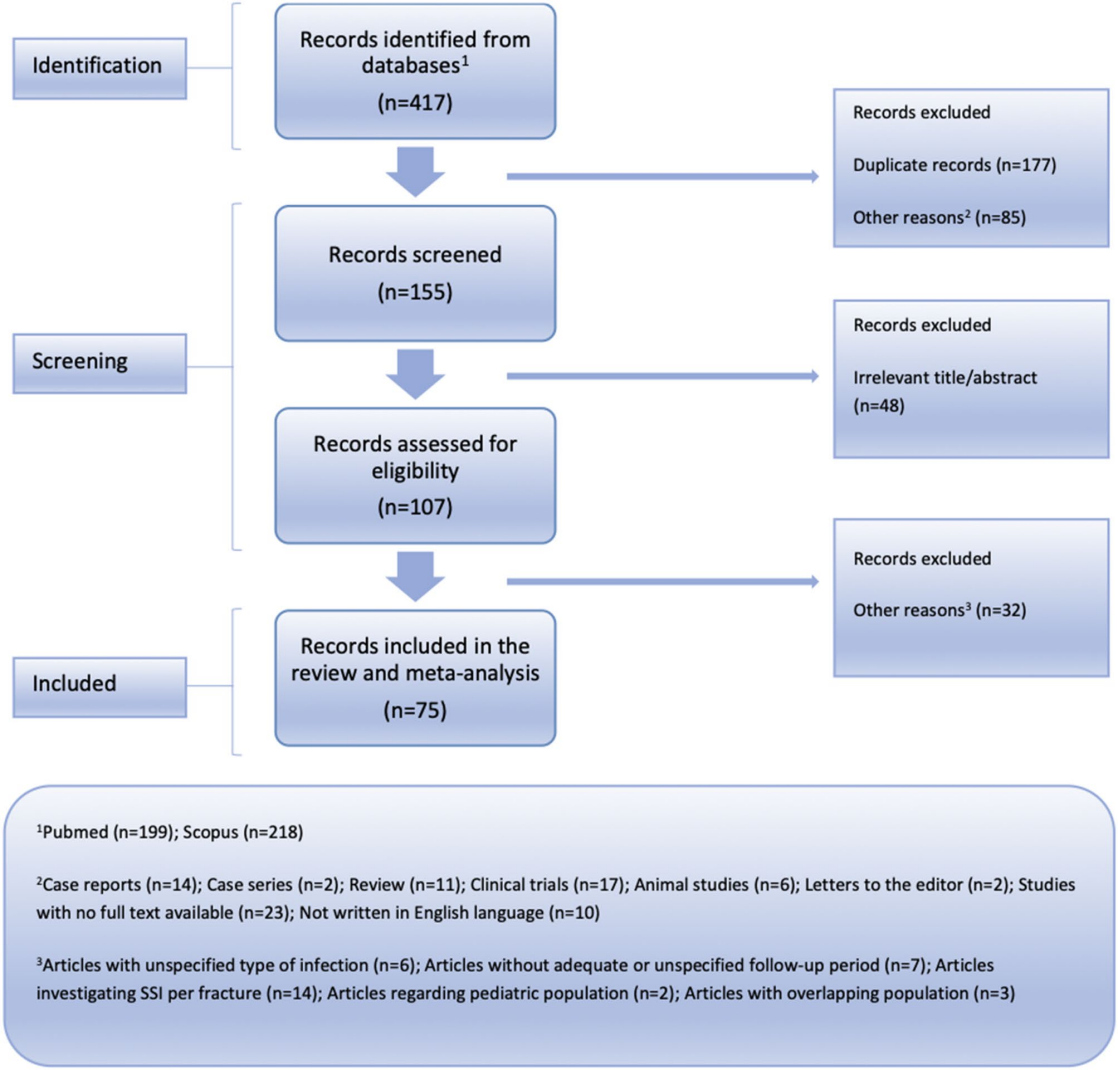
Flow chart depicting the systematic search results from the relevant studies’ identification and selection.

A literature search of Medline (PubMed search engine) and Scopus databases was conducted through an inception up to February 26, 2023, based on the PRISMA guidelines (Fig. 1)^18^. The PRISMA checklist can be found in Supplementary materials (Supplementary Table 1). The literature search was independently performed by two reviewers using a combination of the following keywords: “*mandibular*”, “*mandible*”, “*jaw*”, “*fractures*”, “*open reduction and internal fixation*”, “*ORIF*”, “*surgical site infection*”, “*surgical wound infection*”, “*ssi*”, “*prevalence*”, “*incidence*”, “*rate*”. The reference lists of all identified eligible studies were evaluated for potentially missed articles throughout the initial literature search. Following the aforementioned procedure, all studies were stored in the Zotero reference management software (version 6.0.18) and the duplicate citations were removed^19^. The remaining articles were independently screened by two investigators to identify the studies that met the inclusion criteria. The study selection was conducted in two stages. First, article titles and abstracts were reviewed and those that did not meet the inclusion/exclusion criteria were removed. Secondly, full texts of the remaining articles were retrieved and evaluated. If an absence in studies selection was notified, the final decision was reached by team consensus.

#### Criteria for study selection and data extraction

Articles that examined specifically the prevalence rates of SSI following ORIF procedures for mandibular fractures were included with no restriction on publication date. Case reports, case series with less than ten participants, review articles, randomized clinical trials^20,21^, animals studies, letters to the editor, books, expert opinion, conference abstracts, studies with no full-text available, studies not written in English, articles without adequate (at least one month postoperative) or unspecified follow-up period^22–28^, studies regarding pediatric population^17,29^, studies conducting in Africa^30^, articles with unspecified type of infection, studies regarding non mandibular fractures^31^, studies regarding solely SSI in population with comorbidities (e.g. diabetes melitus, autoimmune diseases), articles containing data derived from surveillance databases and articles investigating postoperative infections per fracture^32–35^ were excluded. In articles with overlapping populations, the most recent or most complete publication was considered eligible. The following variables were obtained from each study: the first author’s name, year of publication, study design, continent of origin, study period, total patients, fractures patients ratio, proportion of males, mean age, and patients with postoperative infections.

#### Quality assessment

Quality appraisal was independently performed by two investigators using the National Heart, Lung, and Blood Institute (NHLBI) Quality Assessment Tools. The NHLBI quality assessment tool for Observational Cohort and Cross-Sectional Studies was employed. Each study was assessed for potential flaws in accordance the methodology or the implementation of each survey that could jeopardize internal validity. For each of the fourteen questions, investigators could select one of the following answers: “yes”, “no”, “cannot determine” (e.g., data were unclear or contradictory) or “not reported” (e.g., missed data) or “not applicable” (e.g., not relevant question regarding this type of study). Study quality was defined as “low”, “moderate” or “high” risk of bias^36^.

#### Statistical analysis

Statistical analysis was carried out using RStudio (version: 2022.12.0 + 353) software (RStudio Team^37^. The meta-analysis was conducted through metafor package^38^. The DerSimonian and Laird random-effects model was used to estimate the pooled prevalence and its respective 95% confidence intervals (CI). Freeman-Tukey double arscine transformation was performed^39^. Heterogeneity presence between studies was evaluated through visual inspection of the forest plot and by using the Cochran’s Q statistic and its respective p value. The Higgins I^2^ statistic and its respective 95% CI were used for quantifying the magnitude of true heterogeneity in effect sizes. An I^2^ value of 0–40%, 30–60%, 50–60% and 75–100% indicated not important, moderate, substantial and considerable heterogeneity, respectively. To determine if the potential outlying effect sizes were also influential, screening for externally studentized residuals with z-values larger than two in absolute value and leave-one-out diagnostics were performed^40^. Due to high heterogeneity remaining, subgroup and meta-regression analysis were performed. In the conducted subgroup analysis, the continent of origin was chosen as the categorical moderator on effect sizes. In the performed meta-regression analysis with continuous variables, the year of publication, the proportion of males and the mean age were assessed as moderators on effect sizes. Due to paucity of data (less than ten studies for each covariate) regarding the smoking status and other variables (e.g., duration of surgery, alcohol, obesity, surgeon level), these data were not included in this analysis^41^. Unless otherwise stipulated, the statistical significance was established at *p* = 0.05 (two-tailed). Tests to evaluate publication bias, such as Egger’s test^42^, Begg’s test^43^ and funnel plots, were developed in the context of comparative data. They assume studies with positive results are more frequently published than studies with negative results, however in a meta-analysis of proportions there is no clear definition or consensus about what a positive result is^44^. Therefore, publication bias in this current meta-analysis was assessed qualitatively.

## Results

### Results and characteristics of the included studies

In total, seventy-five studies (comprising a sum of 5825 participants) were finally included in this analysis. The descriptive characteristics of them are reported in Table 1. All articles were published from 1989 to 2022 (conducted from 1980 to 2020). Eighteen of them were of cohort design and the remaining ones of cross-sectional. Most of the studies were carried out in Asia, followed by America and Europe. The average percentage of males was 83.4% and the mean age of participants ranged from 22.4 to 42 years (median: 29.7 years). As per the quality assessment, eight studies were estimated as high quality^6,7,46,48,52,85,92,102^ and the remaining ones, as moderate (Supplementary materials, Supplementary Table 2).

**Table 1:** Descriptive characteristics of the included studies.

Author	Year of publication	Study design	Continent of origin	Country	Study period	Total patients	Fractures per patient	Proportion of males (%)	Mean age (years)	Infection
Ardary^14^	1989	Cross-sectional	America	USA	1986–1988	71	1.4	88.7	28	8
Sargent^45^	1992	Cross-sectional	America	USA	NA	13	1.2	NA	NA	0
Stone^46^	1993	Cross-sectional	America	USA	1987–1991	129	NA	78.3	NA	15
Zachariades^47^	1995	Cross-sectional	Europe	Greece	1987–1992	191	1.3	83	NA	16
Widmark^48^	1996	Cross-sectional	Europe	Sweden	1990–1992	19	NA	84.2	39.5	1
Herford^49^	1998	Cohort	America	USA	NA	84	NA	84.5	32	4
Eckelt^50^	1999	Cross-sectional	Europe	Germany	1980–1996	230	1.2	78.7	34	3
Moreno^6^	2000	Cross-sectional	Europe	Spain	1993–1996	96	1.6	NA	NA	13
Bolourian^51^	2002	Cohort	America	USA	NA	31	1.4	83.9	27	0
Ellis^52^	2002	Cross-sectional	America	USA	1990–2000	402	1	82	28	75
Kim^53^	2002	Cross-sectional	Asia	Korea	1998–2001	49	1.4	79.6	26.6	4
Suzuki^54^	2004	Cross-sectional	Asia	Japan	1998–2001	14	1	85.7	23.1	0
Guimond^15^	2005	Cross-sectional	America	USA	NA	37	NA	86.4	28.6	2
Barry^55^	2007	Cross-sectional	Europe	Ireland	1998–2004	50	NA	96	22.4	4
Tiwana^56^	2007	Cross-sectional	America	USA	1988–2006	102	NA	NA	NA	3
Zix^57^	2007	Cohort	Europe	Switzerland	NA	20	NA	85	33.9	0
Bell^58^	2008	Cross-sectional	America	USA	NA	75	1.1	84	28.2	4
Biglioli^59^	2009	Cross-sectional	Europe	Italy	2006–2008	33	1.2	69.7	34	2
Bui^60^	2009	Cross-sectional	America	USA	2003–2007	49	NA	90	26	4
Burm^61^	2009	Cross-sectional	America	USA	NA	35	1.7	82.9	30.3	0
Downie^62^	2009	Cohort	Europe	UK	NA	50	1	NA	NA	1
Gerbino^4^	2009	Cross-sectional	Europe	Italy	1998–2008	25	NA	NA	NA	2
Bindra^63^	2011	Cohort	Asia	India	NA	10	NA	100	31.6	0
Chen^64^	2011	Cross-sectional	Asia	Taiwan	1994–2004	51	NA	58.8	28.8	2
Hochuli-Vieira^65^	2011	Cross-sectional	Europe	Germany	2008	45	1	84.5	29	2
Li^66^	2011	Cross-sectional	Asia	China	2001–2006	21	NA	76.2	34.8	2
Benech^67^	2011	Cross-sectional	Europe	Italy	2006–2008	14	1.1	71.4	33	0
Gokkulakrishnan^68^	2012	Cohort	Asia	India	2009–2011	40	NA	NA	31	2
Hofer^69^	2012	Cross-sectional	Europe	Germany	2005–2008	60	NA	86.7	31.1	3
Kim^70^	2012	Cross-sectional	Asia	South Korea	2007–2009	28	NA	89.3	32.4	0
Zhou^71^	2012	Cohort	Asia	China	2006–2009	78	1.3	79.5	31.6	0
Kang^72^	2013	Cross-sectional	America	USA	2010–2011	10	1.8	100	27	0
Lee^73^	2013	Cross-sectional	America	USA	1999–2011	218	NA	75.7	28.3	3
Singh^74^	2013	Cohort	Europe	UK	NA	302	1.4	89.4	NA	29
Pal ^75^	2013	Cross-sectional	Asia	India	NA	18	NA	72.2	NA	1
Rao^76^	2013	Cross-sectional	Asia	India	NA	15	1.1	100	NA	0
Prasad^77^	2013	Cohort	Asia	India	2007–2008	18	NA	NA	NA	3
Yabe^78^	2013	Cross-sectional	Asia	Japan	1997–2012	14	1.1	71.4	28.6	1
Yazdani^79^	2013	Cohort	Asia	Iran	NA	87	NA	83.9	NA	4
Chhabaria^80^	2014	Cohort	Asia	India	NA	20	1.1	90	29	2
Gutta^7^	2014	Cross-sectional	America	USA	NA	363	NA	88	35.5	55
Kanno^16^	2014	Cohort	Asia	Japan	2010–2011	12	NA	75	32.2	0
Song^81^	2014	Cross-sectional	Asia	South Korea	NA	34	NA	79.4	30.3	0
Spinzia^82^	2014	Cross-sectional	Europe	Italy	2003–2011	25	1.1	72	27	1
Pilanci^83^	2014	Cross-sectional	Asia	Turkey	2010–2013	16	1.3	81.3	27	1
Rahpeyma^84^	2014	Cross-sectional	Asia	Iran	2006–2010	25	NA	81.8	41.3	0
Bhatt^85^	2015	Cohort	Asia	India	2007–2010	60	NA	91.7	27.4	5
Pandey^86^	2015	Cohort	Asia	India	2011–2012	15	NA	93	29.6	0
Tracy^87^	2015	Cross-sectional	America	USA	2011–2012	86	NA	91.9	NA	10
Aslan^88^	2016	Cross-sectional	Asia	Turkey	2012–2014	24	NA	66.7	34.6	0
Balaji^89^	2016	Cross-sectional	Asia	India	2004–2014	75	NA	85.3	NA	0
Domingo^90^	2016	Cross-sectional	America	USA	2006–2012	203	NA	NA	NA	33
Odom^91^	2016	Cross-sectional	America	USA	2003–2013	342	NA	86	29.8	32
Spinelli^92^	2016	Cohort	Europe	Italy	2000–2012	389	NA	66.3	28.7	32
Yadav^93^	2016	Cross-sectional	Asia	India	2014–2015	28	NA	86	NA	2
Bouchard^94^	2017	Cross-sectional	America	Canada	2009–2013	78	NA	93.6	25.2	29
Bruneau^95^	2017	Cross-sectional	Europe	Switzerland	2007–2015	43	1.1	86	41.5	0
Monnazzi^96^	2017	Cross-sectional	America	Brazil	1992–2012	149	1	82	26.5	22
Rastogi^97^	2017	Cross-sectional	Asia	India	2013–2016	30	NA	80	29.1	2
Ribeiro-Junior^98^	2017	Cross-sectional	America	Brazil	NA	50	1.1	82	30.6	3
Lim^99^	2017	Cross-sectional	Asia	Korea	2011–2015	49	NA	87.8	NA	5
Ferreira^100^	2018	Cross-sectional	America	Brazil	2011–2015	19	NA	100	27	2
Van Hevele^101^	2018	Cross-sectional	Europe	The Netherlands	2012–2016	53	NA	77.4	42	0
Balasundram^102^	2019	Cross-sectional	Asia	Malaysia	2009–2012	593	NA	88	NA	34
Choi^103^	2019	Cross-sectional	Asia	Korea	NA	14	NA	85.7	36	1
Rao^104^	2019	Cross-sectional	Asia	India	NA	13	1.3	100	28	0
Sudheer^105^	2019	Cross-sectional	Asia	India	2017–2018	10	NA	80	34.7	0
Bhardwaj^106^	2020	Cross-sectional	Asia	India	2016–2019	57	ΝΑ	57.9	NA	3
Bhargava ^107^	2020	Cohort	Asia	India	NA	20	NA	80	NA	0
Felix^108^	2020	Cross-sectional	Asia	India	NA	10	NA	90	NA	1
Ramaraj^109^	2020	Cohort	Asia	India	NA	26	1.2	80.8	37	0
Bhagat^110^	2021	Cross-sectional	Asia	India	NA	12	NA	91.7	36	NA
Singla^111^	2021	Cohort	Asia	India	NA	15	NA	93.3	NA	0
Kumar^112^	2022	Cross-sectional	Asia	India	NA	20	NA	NA	NA	0
Lagana^113^	2022	Cross-sectional	Europe	Italy	2010–2015	13	NA	69.2	28.6	0

*NA* not applicable.

### Prevalence of SSI following ORIF for mandibular fractures

**Figure 2:**
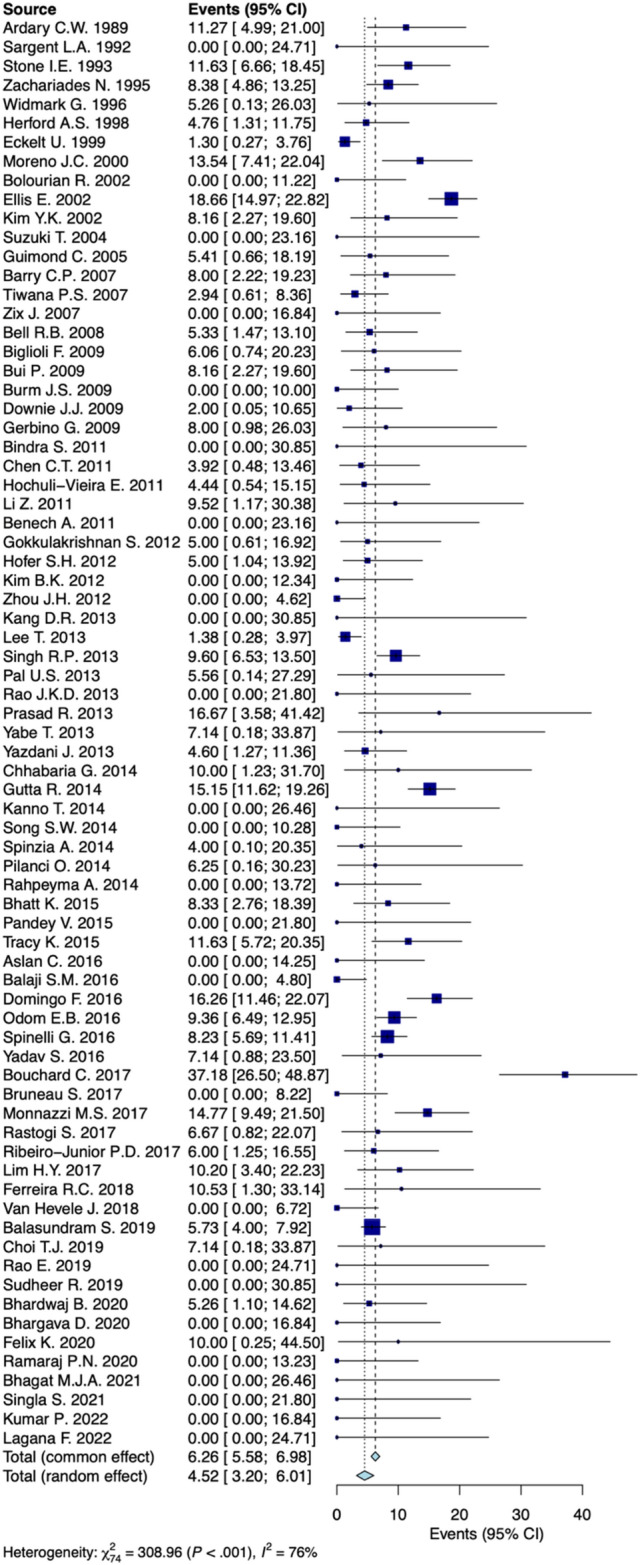
Forest plot evaluating the calculated prevalence of SSI following ORIF for mandibular fractures using random-effects model.

A random-effects model analysis yielded an initial overall SSI prevalence following ORIF of 4.5% (95% CI 3.2–6.0%) with considerable between studies heterogeneity I^2^ = 76% (95% CI 60.5–80.6%, *p* < 0.001) (Fig. 2). The influence diagnostics and the forest plot illustrating the results of the leave-one-out analysis is presented in Supplementary material (Supplementary Fig. 1, Fig. 2). As per them, the study conducted from Bouchard et al. identified as influential. After the exclusion of the aforementioned study the estimated prevalence was calculated at 4.2% (95% CI 3.0–5.6%) with substantial between studies remaining heterogeneity I^2^ = 72.3% (95% CI 51.0–75.0%) (*p* < 0.001).

### Subgroup and meta-regression analysis

The forest plot of the subgroup analysis is illustrated in Supplementary material (Supplementary Fig. 3). The prevalence was 4.2% (95% CI 2.2–6.6%) among studies conducted in Europe, 4.3% (95% CI 3.1–5.6%) among studies conducted in Asia and higher among those conducted in America (7.3%) (95% CI 4.7–10.3%). Heterogeneity remained high in studies conducted in America and moderate in those conducted Europe and low among those conducted in Asia. According to the results of the test for subgroup differences a statistically significant finding was noted. In the meta-regression analysis with continuous variables, a statistically significant negative association between age and the odds of SSI after ORIF for mandibular fractures was observed, as illustrated in Supplementary material (Supplementary Table 1).

## Discussion

To the best of our knowledge this is the first attempt to evaluate the prevalence of SSI after ORIF for mandibular fractures through a systematic review. Therefore, there are no published data available to compare our estimate with. According to the results of this study, the prevalence of SSI following ORIF for mandibular fractures is estimated at 4.2% (95%CI 3.0%-5.6%) with substantial heterogeneity. The subjectivity of the SSI’s diagnosis among medical doctors, the type of surgery and other potential risk factors such as diabetes, prolonged operative time, obesity, patients’ age, gender, additional procedures, smoking status, alcohol consumption, oral hygiene, perioperative warming and the lack of defined guidelines regarding the antibiotic protocol use may influence the prevalence of SSI following ORIF for mandibular fractures^114–119^. Moreover, significant heterogeneity is expected in prevalence and incidence estimates due to the type of this study (differences in the time and place where included studies were conducted). Therefore, high I^2^ in the context of proportional meta-analysis does not necessarily mean that data is inconsistent^44^. In the subgroup analysis, this prevalence varied by the continent of study’s origin, ranging from 4.2% (95% CI 2.2–6.6%) among European studies, to 7.3% (95% CI 4.7–10.3%) among American and 4.3% (95% CI 3.1–5.6%) among Asian ones. Regarding the statistically significant difference in subgroup analysis, due to lack of data, it was not possible to determine the factors associated with it. It is worth emphasizing again that the current studies are observational and have been conducted in different locations, times, and conditions. Additionally, the pathogens that can contaminate surgical wounds, the surgical practices or even the antibiotics used may vary from country to country. Similar, reduction in the prevalence of SSI was found in a recent meta-analysis^120^. However, this study does not provide a comprehensive representation since it utilizes data from multiple and in many cases unidentified surgical procedures. Regarding the influence of the age in meta-regression analysis, it should be noted that, on the one hand, the mean ages range from 22.4 to 42 years, which means that the majority of the patient related comorbidities (e.g., type 2 diabetes, which occurs above the age of 45^121^) commonly associated with aging are likely in their early stages or have not yet become evident, thereby implying that their potential influence on surgical outcomes remains limited. On the other hand, it is important to recognize that this result could be influenced by various confounding factors that require further investigation.

According to WHO in low- and middle-income countries, one out of ten patients (11.8%) develop an SSI after a surgical procedure^122^. ORIF for mandibular fractures can be consider a safe surgical procedure, regardless of the specific nature of this operation. In most SSI, the responsible pathogens originate from the patient's endogenous flora. The oral cavity consists of a unique environment coated with a plethora of bacteria, which form the bacterial biofilm. The oral microbiome can be categorized into two types: the core microbiome, which is common to all individuals and the variable microbiome, which is unique to individuals based on their lifestyle and physiological differences. The normal microbiome is formed by bacteria, fungi, viruses, archaea and protozoa. Among them, the most commonly isolated bacteria are Gram positive Cocci (e.g. Streptococcus, Peptostreptococcus), Gram positive Rods (e.g. Actinomyces, Lactobacillus), Gram negative Cocci (e.g. Moraxella, Veillonella), Gram negative Rods (e.g. Campylobacter, Fusobacterium)^123^. These bacteria are capable of contaminating oral wounds. In terms of the treatment used for mandibular fractures, Jazayeri et al.^124^ analyzed data from nine studies (involving 667 patients) found that ORIF is associated with a higher incidence of postoperative infection (relative risk, 3.6; 95% CI 3.9 to 13.8) compared to closed reduction. Regarding the plate and screw system (locking or nonlocking) used, Zhan et al.^125^ using data from three studies showed no statistical difference in infection rate between groups (Odds Ratio, 0.43; 95% CI 0.13–1.41; *p* = 0.17). And in another study conducted by Khavanin et al.^126^ the author based on the available retrospective studies found out that tooth extraction (which located in line of the fracture) during ORIF procedures was not associated with increased risk of SSI.

Even if the prevalence of SSI may be considered low, SSI still remains one of the most frequent types of health care-associated infections. In order the impact of SSI to be minimized, it is mandatory that necessary preventive measures such as, screening for colonization, isolation of patients with multidrug resistant bacteria, decolonization, surgical site preparation, surgical hand preparation, wearing sterile protective equipment and hygiene and aseptic techniques to be followed^122,127^. To date, there is no consensus regarding the antibiotic regimen used; hence, specific guidelines in country level should be implemented by global organizations, in order inappropriate antibiotic prescribing and the devastating consequences of it, to be avoided. The excess prescription of broad-spectrum chemoprophylaxis leads to antimicrobial resistance, which poses a major threat to public health by increasing mortality around the world, especially in low resources settings. According to a recent systematic review regarding the antibiotics prophylaxis in maxillofacial trauma, preoperative antibiotics were related with lower infection rates while prolonged antibiotic regimens showed no significant benefit^128^. Moreover, de Jonge et al.^129^ combining data from fourteen studies (54,552 participants) found out that the administration of antibiotic prophylaxis for more than 120 min prior to the first incision or after the inception of the surgical procedure was associated with higher risk of SSI than administration less than 120 min. Tetanus prophylaxis should also be considered in open mandibular fractures. Pain control should be achieved with acetaminophen, NSAIDs, and/or opioids. Steroids and ice packs are useful for reducing edema^3^. It is important for healthcare professionals to be aware of the etiology of these infections. Consequently, it is imperative to conduct both prospective and retrospective studies, including observational and interventional approaches, to thoroughly investigate the correlation between SSI following ORIF for mandibular fractures and potential risk factors.

### Study’s strengths and limitations

The main strength of the current study was the comprehensive methodology applied for the literature search, study selection, inclusion/exclusion criteria, screening for eligibility, quality assessment and pooling analysis of prevalence data from forty studies. However, the present study had several limitations. It should be noted that the unidentified heterogeneity remained substantial, therefore, the results should be interpreted with caution. The highly heterogenous outcomes across the included studies were expected due to the nature of this type of studies. The subjectivity of the SSI’s diagnosis among medical doctors and other potential risk factors such as diabetes, prolonged operative time, obesity, patients’ age, gender, additional procedures performed, smoking status, alcohol consumption, oral hygiene, perioperative warming, the type of surgery and the lack of defined guidelines regarding the antibiotic protocol used might bias the prevalence of SSI following ORIF for mandibular fractures. Due to limited data (less than ten studies for each covariate) regarding variables such as smoking status, duration of surgery, alcohol, obesity, surgeon level, these variables were excluded from this presented analysis. Moreover, only observational studies written in English language were included resulting in the occurrence of reporting bias. Consequently, the existing evidence may be constrained and lacking comprehensive representation due to the omission of studies composed in languages other than English (e.g. studies carried out and documented in countries where English is not the primary language and which possess limited resources). Only studies from Europe, America, and Asia were finally included in our analysis. Therefore, it is important to note again that the results should be interpreted cautiously due to the limited generalizability of the data and the potential underestimation or overestimation of the prevalence.

## Supplementary Information

Supplementary Information.

## Data Availability

Literature and Rstudio data are available from the corresponding author on reasonable request.
